# Invasive Group A Streptococcal Disease: Risk Factors for Adults

**DOI:** 10.3201/eid0908.020745

**Published:** 2003-08

**Authors:** Stephanie H. Factor, Orin S. Levine, Benjamin Schwartz, Lee H. Harrison, Monica M. Farley, Allison McGeer, Anne Schuchat

**Affiliations:** * Centers for Disease Control and Prevention, Atlanta, Georgia, USA; † New York Academy of Medicine, New York, New York, USA; ‡ National Institute for Allergy and Infectious Diseases, Bethesda, Maryland, USA; § Johns Hopkins University, Baltimore, Maryland, USA; ¶ Emory University, Atlanta, Georgia; # Mount Sinai Hospital**,** Toronto, Ontario, Canada

**Keywords:** *Streptococcus pyogenes*, group A streptococcal disease, invasive disease, risk factors, case-control, sequential-digit dialing, necrotizing fasciitis, streptococcal toxic shock syndrome, conditional logistic regression, research

## Abstract

We conducted a case-control study to identify risk factors for invasive group A streptococcal (GAS) infections, which can be fatal. Case-patients were identified when *Streptoccus pyogenes* was isolated from a normally sterile site and control subjects (two or more) were identified and matched to case-patients by using sequential-digit telephone dialing. All participants were noninstitutionalized surveillance area residents, >18 years of age. Conditional logistic regression identified the risk factors for invasive GAS infection: in adults 18 to 44 years of age, exposure to one or more children with sore throats (relative risk [RR]=4.93, p=0.02), HIV infection (RR =15.01, p=0.04), and history of injecting drug use (RR=14.71, p=0.003); in adults >45 years of age, number of persons in the home (RR=2.68, p=0.004), diabetes (RR= 2.27, p=0.03), cardiac disease (RR=3.24, p=0.006), cancer (RR= 3.54, p=0.006), and corticosteroid use (RR=5.18, p=0.03). Thus, host and environmental factors increased the risk for invasive GAS disease.

Invasive GAS infection can lead to dramatic, rapidly-progressive syndromes such as necrotizing fasciitis and streptococcal toxic shock syndrome (STSS). An estimated 9,500 cases of invasive GAS disease occurred in the United States in 1999, resulting in approximately 1,100 deaths. The overall case-fatality rate of invasive GAS is estimated to be from 10% to 15%, and the case-fatality rate for STSS can exceed 60% (*1*). Most of these infections are community-acquired.

Although case series and population-based surveillance have identified several possible host risk factors for the development of invasive GAS disease, including age, Native American ethnicity, HIV infection, diabetes mellitus, cardiovascular disease, alcoholism, and other chronic diseases (*1*–*3*), these studies have not been able to assess household risk factors. In addition, surveillance studies identified possible risk factors by comparing prevalence of host factors among GAS patients (by using medical record data) to prevalence of host factors among the general population using population-based estimates (*1*,*2*). These comparisons are limited by the completeness and availability of both kinds of data.

Household-based studies conducted in the 1950s demonstrated that school-aged children were most often responsible for introducing a GAS strain into a household and that mothers were more likely to subsequently acquire the bacteria than fathers (*4*). These studies suggest that exposure to children and duration of exposure to a GAS-infected person influence the transmission of GAS within households. However, community studies have not shown the relative importance of these factors compared to host factors.

We conducted a case-control study to evaluate the importance of previously identified risk factors for invasive GAS infection: contact with other persons in the home, with children, with persons symptomatic with GAS disease in the home, with other persons at work, and with persons symptomatic with GAS disease at work.

## Methods

Invasive GAS disease was defined as the isolation of *Streptcoccus pyogenes* from a normally sterile site (e.g., blood, cerebrospinal fluid, pleural fluid, peritoneal fluid, pericardial fluid, joint fluid, surgical specimens, bone, and scrotal fluid) in a noninstitutionalized resident, >18 years of age, in a surveillance area. Persons who had group A *Streptococcus* isolated from a sterile site more than 48 hours after hospital admission were presumed to have a nosocomial infection (*5*) and were excluded.

Cases of invasive GAS disease were identified through active, laboratory-based surveillance in three areas: metropolitan Atlanta, Georgia, from July 1, 1997, through June 30, 1999; metropolitan Baltimore, Maryland, from July 1, 1997, through June 30, 1999, and the Toronto-Peel region, Ontario, Canada, from July 1, 1997, through December 31, 1997. The surveillance area population was estimated to include 9 million people (3,627,184 in metropolitan Atlanta, 2,436,239 in the Baltimore metropolitan area, and 3,008,570 in the Toronto-Peel region based on 1997 Bureau of Census estimates [*6*]). All acute-care hospitals and laboratories serving the residents of the surveillance area were contacted biweekly and audited semiannually to identify patients with invasive GAS disease.

A “case algorithm” was used to contact persons infected with invasive GAS infection. For each case-patient identified, up to 15 separate telephone calls were made to contact the patient. To maximize the likelihood of contacting the case-patient, the telephone calls were made on 5 nonconsecutive days, including at least 1 weekend day, during each of three different time periods (8 a.m.–noon; noon–5 p.m.; 5 p.m.–8 p.m.). The party was considered unavailable for that call if the telephone was allowed to ring 10 times or if an answering machine picked up the phone. Persons still hospitalized when the case was identified were interviewed by phone in the hospital when feasible. Case-patients were eligible if their enrollment was complete within 3-months of onset of their GAS disease.

To both maximize enrollment and limit bias, several arrangements were made. Family members of deceased case-patients were interviewed. Non–English-speaking patients were included if the individual surveillance sites had the resources to communicate with the patients in their language.

A population-based sample of matched control subjects was selected through a process of systematic, sequential-digit telephone dialing. Case-patients and control subjects were matched by age group, postal or zip code, and telephone exchange. Age groups were defined as follows: 18–44 years of age, 45–64 years of age, and >65 years of age. Matching was done on postal or zip code and telephone exchange to control for socioeconomic status. Because control-subjects were identified by telephone interview, case-patients who did not have a telephone were excluded.

A “control algorithm” was used to identify control subjects. Control identification began after a case-patient had been identified, confirmed as eligible, and enrolled in the study. Phone numbers for control subjects were generated by incrementally adjusting the enrolled case-patient’s home phone number. For example, if an enrolled case-patient’s telephone number ended in 1234, the first phone number used to identify a control had the same area code and exchange and ended in 1235. The numbers generated for control subjects were called a maximum of four times over several days. Any number of phone numbers were called at one time. Calling continued until 1) a minimum of two control subjects were enrolled per case-patient, and 2) all numbers that had been called at least once completed the control algorithm. As a result, more than two control subjects were enrolled for some case-patients. The purpose of completing the control algorithm was to ensure that the control group was not biased towards households that were easier to contact.

When experienced surveillance personnel reached the case-patient or control subject, they explained the purpose of the study, obtained informed consent, and administered a standardized questionnaire. This study was approved by the Institutional Review Boards at the Centers for Disease Control and Prevention and at each of the sponsoring institutions.

### Questionnaire

The questionnaire included demographic information, socioeconomic status, smoking status of the interviewee, smoking status of other persons in the home, medical history, and history of alcohol and injecting drug use. Within the medical history section, we differentiated between “regular” nonsteroidal antiinflammatory drug (NSAID) use and “new” NSAID use. New NSAID use implied that the case-patient had started using NSAIDs in the 2 weeks before their illness or that a control subject had started using NSAIDs in the 2 weeks before their interview. Each case-patient and control subject were allowed to self-define “regular use” of NSAIDS. To screen for alcoholism, we used the CAGE questionnaire in which a subject is asked four questions about his or her relationship to alcohol. The likelihood of alcoholism increases with the number of times the person answers “Yes” (*7*).

The questionnaire also asked about contact with other persons in the home, contact with children, contact with persons exhibiting symptoms of GAS disease in the home, contact with other persons at work, and contact with persons with symptoms of GAS disease at work. To evaluate contact with other persons in the home, the questionnaire included questions about number of persons in the home, crowding in the home (number of persons per room), type of home, age of all persons living in the home, and types of relationships with all persons living in the home. To evaluate contact with children, the questionnaire asked about the number of children in the home (i.e., persons <18 years of age), amount of time spent with children in the home, frequency of certain types of sharing behavior with children in the home (including eating off the same plate, sharing a beverage, sleeping in the same bed), amount of time spent with children who did not live in the home, and frequency of sharing behavior with children who did not live in the home.

The questionnaire asked whether anyone in the home had been ill in the 2 weeks before the case-patient’s illness or the control subject’s interview by asking about specific types of illnesses. For example, the questionnaire asked “Has anyone in your household, other than yourself, had a sore throat?” If the interviewee said “yes,” he or she was asked how many children and how many adults had a sore throat during that period. The questions asked whether anyone in the home had a sore throat; fever; new cough; new runny nose; skin infection; a diagnosis of strep throat, an ear infection, or a sinus infection; or died. To measure the severity of the illness, the questionnaire asked whether the ill person(s) had visited a doctor, missed school or work, had taken antibiotics, or had been hospitalized.

To evaluate exposure to both persons at work and symptomatic persons at work, the questionnaire asked which type of environment the case-patient or control subject worked in, how many hours she or he worked, how many persons were in their work environment, and whether anyone at work had been ill in the 2 weeks before the case-patient’s illness or control subject interview by asking about specific types of illnesses. The questions were asked in the same manner as those asked of persons in the home.

### Statistical Analysis

Odds ratios (ORs) for each potential risk factor were determined by using conditional logistic regression (Proc PHREG, SAS Version 6.12, Cary, NC), controlling for sex and race. These ORs were determined separately for age groups 18–44 years of age and >45 years of age to identify age-dependent risk factors. Those variables found to have a p value >0.20 in individual analyses were included in multivariable analysis. Computer-assisted and manual forward, backward, and stepwise conditional logistic regression was done to identify risk factors independently associated with invasive GAS disease. ORs with 95% confidence intervals (CIs) that do not include 1.00, and p values <0.05 were considered statistically significant in multivariable analysis.

## Results

Surveillance identified 401 episodes of invasive GAS disease among adults >18 years of age; 390 persons were traceable and were screened by surveillance personnel for possible participation. Of the 390 persons with invasive GAS disease, 49 were ineligible (36 due to nosocomial infection and 13 due to institutionalization). Of the remaining 341 case-patients, 139 were enrolled, 24 refused to participate, 27 were not reached after exhausting the telephone case algorithm, and the rest were nonparticipants. Reasons for nonparticipation included the following: refusal to allow participation by spouse or surrogate, >3 months had elapsed since the illness, incomplete or incorrect contact information (i.e., wrong phone number, disconnected phone, no phone, homelessness), and difficulty with communication over the phone (i.e., poor communication skills, non–English-speaking patient).

The number of case-patients and control subjects enrolled varied by area: 58 case-patients were from Atlanta, 49 case-patients were from Baltimore, and 32 case-patients were from Toronto. Of the 139 case-patients enrolled, 48 (34%) were 18–44 years of age, 54 (39%) were 45–64 years of age, and 37 (27%) were >65 years of age. Of the 139 case-patients, 70 (50%) were men, 75 (54%) were white, and 60 (43%) were African-American (Table 1). Eighteen (13%) of 139 patients died. Primary bacteremia and cellulitis were the two most common diagnoses (Table 2). The organism was most commonly identified from blood.

**Table 1:** Characteristics of patients with invasive group A streptococcal disease, Atlanta, Baltimore, Toronto, 1997–1999

Characteristic	Patients N=139 (%)
Geographic area	
Atlanta	58 (42)
Baltimore	49 (35)
Toronto	32 (23)
Age group	
18–44	48 (34)
45–64	54 (39)
65+	37 (27)
Sex	
Male	70 (50)
Female	69 (50)
Race	
White	75 (54)
Black	60 (43)
American Indian or Alaskan Native	1 (1)
Asian or Pacific Islander	2 (1)
Other or not specified	1 (1)
Ethnicity	
Hispanic	2 (1)
Non-Hispanic	137 (99)
Outcome	
Lived	120 (86)
Died	18 (13)
Unknown	1 (1)

**Table 2:** Clinical syndromes of patients with invasive group A streptococcal disease, Atlanta, Baltimore, Toronto, 1997–1999

Clinical syndromes^a^	Patients N=139 (%)
Primary bacteremia (without focus)	57 (41)
Cellulitis	36 (26)
Septic arthritis	11 (8)
Necrotizing fasciitis	9 (6)
Pneumonia	8 (6)
Streptococcal toxic shock syndrome	6 (4)
Otitis	4 (3)
Peritonitis	3 (2)
Osteomyelitis	2 (2)
Endometritis	2 (2)
Abscess	2 (2)
Puerperal sepsis	2 (2)
Meningitis	1 (1)
Endocarditis	1 (1)
Urologic syndrome	1 (1)
Other syndrome	18 (13)

^a^Patients may appear in more than one category.

Several factors were associated with invasive GAS disease among those 18–44 years of age (Table 3). When sex and race were controlled for, HIV seropositivity and history of injecting drug use were significantly associated with invasive GAS disease (p<0.05) when each variable was analyzed individually. Smoking, presence of children in the home, diabetes, cancer, regular use of NSAIDs, corticosteroid use, and alcohol abuse were not associated with invasive GAS disease. Using multivariable conditional logistic regression and controlling for sex and race, we found that three risk factors were independently associated with invasive GAS disease: having one or more children with a sore throat in the home in the past 2 weeks (RR=4.93, p=0.02), HIV seropositivity (RR=15.01, p=0.04), and history of injecting drug use (RR=14.71, p=0.003). Among those 18–44 years of age, one case-patient had a history of paralysis, and no one had recent varicella infection.

**Table 3:** Individual risk factor and multivariable analysis for risk factors for invasive group A streptococcal disease among case-patients and control subjects matched on age and zip code, 18–44 years of age, Atlanta, Baltimore, Toronto, 1997–1999^a,b,c^

			Univariate analysis		Multivariable analysis	
Variable	Case-patients (n=48)	Control subjects (n=115)	OR (95% CI)	p value	OR (95% CI)	p value
Number of persons living in the home						
3+ 1–2	38 (79) 10 (21)	80 (70) 35 (30)	1.91 (0.81 to 4.47)	0.14		
Smoke expo- sure						
Current smoker with passive smoke exposure	6 (13)	17 (15)	1.04 (0.32 to 3.37)	0.95		
Current smoker without passive smoke exposure	12 (25)	16 (14)	2.35 (0.85 to 6.48)	0.86		
Passive smoke exposure	6 (13)	12 (10)	1.82 (0.57 to 5.82)	0.31		

No smoke exposure	24 (50)	70 (61)	1.00			
Any child <18 years living in the home						
Yes No	30 (63) 18 (37)	68 (59) 47 (41)	1.25 (0.59 to 2.64)	0.57		
>1 Child with sore throat in the family						
Yes No	7 (15) 41 (85)	7 (6) 108 (94)	2.75 (0.87 to 8.70)	0.09	4.93 (1.24 to 19.68)	0.02
Diabetes mellitis						
Yes No	6 (13) 40 (87)	5 (4) 107 (96)	2.11 (0.63 to 7.10)	0.23		
Cancer						
Yes No	0 47 (100)	3 (3) 108 (97)	0	0.99		
Varicella						
Yes No	0 48 (100)	0 115 (100)				
Cirrhosis						
Yes No	0 48 (100)	0 115 (100)				
Paralysis						
Yes No	1 (2) 47 (98)	0 115 (100)				
Regular NSAID use						
Yes No	11 (26) 32 (74)	31 (28) 78 (72)	.97 (0.42 to 2.23)	0.93		
New use of NSAIDs						
Yes No	9 (19) 34 (81)	10 (9) 100 (91)	2.15 (0.79 to 5.80)	0.13		
Use of cortico- steroids						
Yes No	0 44 (100)	3 (3) 109 (97)	0	0.99		
HIV+						
Yes No	7 (15) 39 (85)	1 (1) 110 (99)	12.66 (1.47 to 108.92)	0.02	15.01 (1.09 to 207.30)	.04
Ever injected drugs						
Yes No	10 (22) 36 (78)	3 (3) 110 (97)	11.80 (2.46 to 56.66)	0.002	14.71 (2.52 to 85.70)	.003
Alcohol use (based on CAGE questions)^d^						
CAGE score 0 CAGE score 1 CAGE score 2 CAGE score 3 CAGE score 4	28 (72) 5 (13) 0 5 (13) 1 (3)	57 (68) 22 (26) 3 (4) 2 (2) 0	1.14 (0.69 to 1.89)	0.62		

^a^OR, odds ratio; CI, confidence interval; NSAID, nonsteroidal antiinflammatory drug. ^b^Due to missing data, for some variables, data for fewer than 48 case-patients and 115 control subjects were available. ^c^Analyses controlled for race and sex. ^d^Source (*7*).

Many factors were associated with invasive GAS disease in adults >45 years of age (Table 4). When the results were controlled for sex and race, we found that the following factors were significantly associated with invasive GAS disease (p≤0.05): three or more persons living in the home, any child living in the home, other smokers living in the home, diabetes mellitus, cardiac disease, chronic obstructive pulmonary disease (COPD), cancer, paralysis, regular use of NSAIDs, use of corticosteroids, and history of injecting drug use. Current smoking and alcohol abuse were not associated with invasive GAS disease. When multivariable conditional logistic regression analysis, controlling for sex and race, was used, the following factors were independently associated with GAS disease: three or more persons living in the home (RR=2.68, p=0.004), diabetes mellitus (RR=2.27, p=0.03), cardiac disease (RR=3.24, p=0.006), cancer (RR=3.54, p=0.006), and use of corticosteroids (RR=5.18, p=0.03).

**Table 4:** Individual risk factor and multivariable analysis for risk factors for invasive group A streptococcal disease case-patients and control subjects, matched by age group and zip code, 45+ years old, Atlanta, Baltimore, Toronto, 1997–1999^a,b,c^

Variable			Univariate analysis					Multivariable analysis	
	Case-patients (n=91)	Control subjects (n=196)	OR (95% CI)		p value			OR (95% CI)	p value
Number of persons living in the home									
3+ 1–2	47 (52) 44 (48)	55 (28) 140 (72)	2.64 (1.46 to 4.75)		0.001			2.68 (1.37 to 5.28)	0.004
Any child living in the home									
Yes No	31 (34) 60 (66)	33 (17) 163 (83)	2.12 (1.11 to 4.05)		0.02				
Smoke exposure Current smoker with passive smoke exposure									
	15 (16)	16 (8)	2.37 (0.94 to 6.01)		0.07				
Current smoker without passive smoke exposure							
	12 (13)	32 (16)	1.07 (0.49 to 2.33)	0.86			
Passive smoke exposure									
	19 (21)	25 (13)	2.34 (1.08, 5.04)		0.03				
No smoke exposure									
	45 (49)	123 (63)	1.00						
Diabetes mellitus									
Yes No	28 (31) 62 (69)	32 (16) 164 (84)	2.33 (1.22 to 4.45)		0.01			2.27 (1.07 to 4.81)	0.03
Hypertension									
Yes No	47 (52) 42 (47)	86 (45) 107 (55)	1.61 (0.92 to 2.82)		.10				
Cardiac disease									
Yes No	30 (33) 60 (67)	24 (12) 172 (88)	3.09 (1.58, 6.06)		0.001			3.24 (1.40 to 7.51)	0.006
Chronic obstructive pulmonary disease (COPD)									
Yes No	8 (9) 79 (91)	2 (1) 193 (99)	7.85 (1.50 to 41.07)		0.01				
Cancer									
Yes No	18 (20) 71 (80)	22 (11) 173 (89)	2.58 (1.23 to 5.41)		.01			3.54 (1.44 to 8.70)	0.006
Varicella									
Yes No	1 (1) 85 (99)	0 191 (100)	Undefined		.99				
Cirrhosis									
Yes No	5 (6) 82 (94)	0 195 (100)	Undefined		0.99				
Paralysis									
Yes No	5 (6) 84 (94)	1 (1) 195 (99)	11.61 (1.34 to 100.87)		0.01				
Regular NSAID use									
Yes No	43 (48) 46 (52)	64 (34) 125 (66)	1.82 (1.06 to 3.14)		0.03				
New use of NSAIDs									
Yes No	14 (16) 72 (84)	14 (7) 178 (93)	2.13 (0.89 to 5.10)		0.09				
Use of cortico- steroids									
Yes No	9 (10) 77 (90)	3 (2) 193 (98)	4.96 (1.26 to 19.62)		0.02			5.18 (1.14, 23.54)	.03
HIV+									
Yes No	1 (1) 90 (99)	2 (1) 194 (99)	.51 (0.04 to 6.14)		0.59				
Ever injected drugs									
Yes No	6 (7) 78 (93)	3 (2) 190 (98)	5.48 (1.00 to 30.09)		0.05				
Alcohol use based on CAGE questions									
CAGE score 0 CAGE score 1 CAGE score 2 CAGE score 3 CAGE score 4	42 (78) 5 (9) 2 (4) 3 (6) 2 (4)	107 (77) 19 (14) 9 (6 ) 4 (3) 0	0.95 (0.56 to 1.61)		.86				

^a^OR, odds ratio; CI, confidence interval; NSAID, nonsteroidal anti-inflammatory drug; ^b^Due to missing data, for some variables, data for fewer than 91 case-patients and 196 control subjects were available. ^c^Analyses controlled for race and sex.

Among adults >45 years of age, the only cases of cirrhosis and recent varicella infection occurred in case-patients; five case-patients reported a history of cirrhosis, and one case-patients reported a recent varicella infection. Five (83%) of six persons with paralysis were in the case-patient group. This difference was significant when this variable was evaluated alone but did not reach statistical significance in multivariable analysis. None of the six patients with paralysis in this age group reported the presence of a decubitus ulcer; two of the persons with paralysis indicated that they had open sores in other places.

Of the 47 patients who had a cutaneous focus of invasive GAS infection (36 with cellulitis, 9 with necrotizing fasciitis, and 2 with an abscess), 39 (83%) reported an open sore, bruise, or burn before the onset of invasive GAS symptoms, and 5 (11%) reported having been diagnosed with a skin condition in the past (eczema, psoriasis, or seborrheic dermatitis).

Type of work environment, number of persons at work, number of hours at work, and presence of an ill person at work were not associated with invasive GAS infection in either age group.

## Discussion

This study suggests that both host and environmental factors are associated with the risk of community-acquired invasive GAS disease in adults. We found that the host factors of HIV infection, diabetes, malignancy, injecting drug use, and cardiac disease were associated with an increased risk of invasive GAS disease. The environmental factors associated with an increased risk were household size and the presence of a child with a sore throat. These environmental factors highlight the importance of person-to-person transmission of group A streptococcus.

Although previous studies have found host risk factors to be associated with invasive GAS infection, this study found that the importance of host factors varied significantly by patient’s age. In adults 18–44 years of age, HIV infection and history of injecting drug use were associated with invasive GAS disease. The association with HIV has been previously identified (*1*) and suggests that immune suppression increases the risk for invasive GAS disease. The association with injecting drug use has been previously identified also (*8*) and may be because of the direct injection of group A streptococci from the skin into the blood. The increased risk associated with injecting drug use was independent of that related to HIV infection. Because control subjects identified by random digit dialing may be reluctant to admit certain illicit behaviors and HIV infection, our ascertainment of injecting drug use and HIV infection among controls may be an underestimate; the strong association between these two factors and invasive GAS infection found in our study may be an overestimate of the true value of this association.

In older adults, diabetes mellitus, cardiac disease, cancer, and corticosteroid use are associated with invasive GAS infection. The association with diabetes, cancer, and corticosteroids again suggests that immune dysfunction is important in the development of this disease. An association between cardiac disease and invasive GAS infection was also suggested in a recent 10-year population-based surveillance study done in the San Francisco Bay area (*8*). The mechanism by which cardiac disease increases the risk of invasive GAS disease is not known but warrants further study.

Our data also suggest that paralysis, cirrhosis, and varicella infections may be risk factors for invasive GAS disease. These conditions were not common among our study participants; thus, the power of this investigation to identify them as significant risk factors was limited. Future research into the association between invasive GAS disease and paralysis should include evaluation of the possible contribution of skin disruption. Varicella infection is a well-documented risk factor in the development of invasive GAS disease among children (*1**,**9**–**14*).

We did not find an independent association between invasive GAS and use of NSAIDs. The hypothesis that NSAID use might increase the risk of necrotizing fasciitis in children with varicella was first suggested by Brogan et al. (*11*), and later observations by Peterson et al. supported it (*12*). Several mechanisms by which NSAID use might influence the incidence or severity of GAS infections have been proposed (*15*). A recent prospective, multicenter case-control study did not find that NSAID use increases the risk of necrotizing GAS infections (*16*). Instead, their data suggested that children with varicella were apt to take NSAIDS because they were ill from varicella as opposed to NSAIDS being a risk factor for later acquisition of invasive GAS disease.

A large proportion of the patients with invasive GAS disease had a cutaneous form of the disease, and a large portion of those with cutaneous disease reported an open sore, bruise, or burn before the onset of invasive GAS symptoms. This suggests the skin is an important portal of entry for invasive GAS infection.

Other studies have found an association between invasive bacterial diseases and cigarette smoking (*17*,*18*). We did not find this association. A true association may have been undetectable because of the difficulty in ascertaining smoking status over the telephone.

Other studies have found an association between invasive GAS disease and alcoholism (*1*). We did not find this association. The sensitivity of the CAGE questions (*7*) may be lower in phone interviews than in face-to-face interviews.

Our study found that environmental exposures were important in the development of invasive GAS infection. Specifically, we found that environmental exposures are related to age. In younger adults, exposure to school-aged children with a sore throat increased the risk of invasive GAS disease. Household studies in the 1950s showed that school-aged children are most likely to introduce group A streptococcus into a household and that symptomatic children are more efficient transmitters of infection than are asymptomatic children (*4*). Surveillance in Toronto demonstrated that the risk of colonization in the household is associated with younger age and 4 or more hours of contact with the infected person per day (*1*). More recently, the role of transmission among household contacts has been demonstrated in studies of community GAS outbreaks and household contacts of patients with invasive GAS (*19*,*20*). These studies suggest that infections among children may represent an important reservoir for infections in adults.

This study has several limitations. The statistical power of this study to detect the association between GAS infection and certain known risk factors, such as concomitant varicella infection, was limited. Because we matched subjects in terms of socioeconomic status, we were unable to examine socioeconomic variables in our analysis. This study included only people who have phones, and risk factors for invasive GAS disease may differ between people who do not have phones and those who do.

The results from this risk factor study can shape current and future strategies to prevent invasive GAS disease. Currently, these findings may help refine recommendations for prophylaxis of close contacts of persons with invasive GAS disease. Vaccines for GAS disease are being developed. Identifying those at greatest risk of invasive GAS disease is important in developing vaccination recommendations. Our data suggest that vaccination should be considered for those with history of injecting drug use, HIV infection, diabetes mellitus, cardiac disease, cancer, and use of corticosteroids. Our data also suggest that vaccination will provide benefits to those who receive the vaccine and to those with whom they live.
